# The effects of the secondary metabolites of the citrus endophytic fungus *Nemania* sp. LJZ-Y-11 on the citrus canker disease-causing pathogen *Xanthomonas citri* subsp. *citri*

**DOI:** 10.1186/s12866-025-04403-8

**Published:** 2025-10-21

**Authors:** Xueyu Chen, Qicong Li, Jie Yao, Linfang Huang, Rourou Lu, Zhiyong Deng, Haiyu Luo, Yecheng Deng, Xianglin Xu

**Affiliations:** 1 Key Laboratory of Ecology of Rare and Endangered Species and Environmental Protection, Ministry of Education-Guangxi Key Laboratory of Landscape Resources Conservation and Sustainable Utilization in Lijiang River Basin, Guangxi Normal University, Guilin Guangxi, 541006 China; 2 University Engineering Research Center of Bioinformation and Genetic Improvement of Specialty Crops, Guangxi Normal University, Guilin, 541006 Guangxi China; 3 Guangxi Key Laboratory of Rare and Endangered Animal Ecology, Guangxi Normal University, Guilin, 541006 Guangxi China; 4 Key Laboratory of Industrialized Processing and Safety of Guangxi cuisine, Guilin Tourism University, Guilin Tourism University, Guilin, 541006 Guangxi China; 5 School of food and health, Guilin Tourism University, Guilin, 541006 Guangxi China

**Keywords:** Active compounds, Antibacterial activities, Growth curves, Citrus canker lesion

## Abstract

The bacterium *Xanthomonas citri* subsp. *citri* (Xcc), which causes citrus canker disease, has caused great harm to citrus production worldwide. In this study, we aimed to clarify the antibacterial activities of extracts prepared with solvents of different polarities from the fungus *Nemania* sp. LJZ-Y-11 against Xcc, isolate and identify the active secondary metabolites, as well as evaluate the effects of the active compound on the Xcc. The results showed that extracts displayed significant antibacterial activities with the minimum inhibitory concentrations (MICs) and the minimal bactericidal concentrations (MBCs) at 0.3125 − 5 mg.mL^−1^ and 0.625 − 5 mg.mL^−1^, respectively. Especially the ethyl acetate (EtOAc) extract displayed the highest activity (MIC = 0.3125 mg.mL^−1^, MBC = 0.625 mg.mL^−1^). Five known compounds (**1** − **5**) were obtained from the EtOAc extract of *Nemania* sp. LJZ-Y-11, and identified as chrysogeside D (**1**), 2-pyruvoylaminobenzamide (**2**), 4-hydroxybenzaldehyde (**3**), stigmasta-7,22-diene-3β,5ɑ,6α-triol (**4**), and (2*S*,5*R*)-2-ethyl-5-methylhexanedioic acid (**5**) based on spectroscopic analysis. Among them, compounds **3** and **5** showed significant inhibitory effects against Xcc, with MICs at 0.25 and 0.125 mg.mL^−1^, respectively, and compound **5** showed more anti-Xcc potential with the MBC of 0.25 mg.mL^−1^. The effects of compound **5** on the growth and cell morphology were dose-dependent and could reduce the formation of lesions or no lesions in leaves. Overall, the results indicated that citrus endophytic fungi could produce active products against the citrus canker pathogen, both crude extracts and compounds of the endophytic fungus *Nemania* sp. LJZ-Y-11 showed good anti-Xcc potential and encouraged further investigation of active compounds produced by citrus endophytic fungi.

**Supplementary Information:**

The online version contains supplementary material available at 10.1186/s12866-025-04403-8.

## Introduction

Endophytic fungi (EF) are an important part of plant-microbe ecosystems; they colonize internal host tissues or organs during parts or all of their life cycles without causing obvious disease symptoms in the host plant [1]. EF can enhance the disease resistance of their hosts [2], and some EF can also produce active antibacterial compounds through artificial culture and fermentation, offering the possibility for large-scale production of these plant disease-control agents. EF and their secondary metabolites have been thought to play a key role in controlling a variety of plant diseases and may serve as environmentally sustainable resources for plant disease control [2, 3].

Citrus canker is a bacterial disease caused by the gram-negative bacterium *Xanthomonas citri* subsp. *citri* (Xcc) and affects all commercial citrus varieties; there is no known cure for citrus canker [4]. Copper-based bactericides are the typical method of control for this disease. However, the long-term and large-scale use of these bactericides results in chemical residues on the fruit and the environment as well as the development of chemical-resistant Xcc [5, 6]. For sustainable citrus production, more effective and safe alternatives to copper-based bactericides are required to control citrus canker, and searching for natural antibacterial active products from plants, animals, or microorganisms, has become an important approach for plant disease control [7–12].

There have already been some reports on the use of secondary metabolites of citrus endophytic fungi to control citrus diseases. For example, it was reported that volatile organic compounds produced by the endophyte *Muscodor* sp. LGMF1254, which was extracted from the leaves of *Citrus sinensis* (Parana´, Brazil), could inhibit the growth of *Phyllosticta citricarpa* (the pathogen responsible for the citrus black spot) [13]. It was also found that the disease incidence and severity of citrus black spot were significantly reduced when fruits were pre-inoculated with the endophyte *P. capitalensis* or *P. paracapitalensis* 14 days before pathogen *P. citricarpa* inoculation into the fruits [14]. The endophyte *Fusarium avenaceum* Gds-1 isolated from citrus fruits has a highly effective and stable biocontrol effect on the citrus blue mold-causing pathogen *Penicillium italicum* both *in vivo* and *in vitro* [15]. The endophyte *Aspergillus aculeatus* GC-09, isolated from a citrus plant called the Gannan navel orange tree, exhibited antifungal activity against *P. italicum* involving the disruption of cell membrane integrity, accumulation of ROS, and reduction of antioxidant enzyme activity [16].

Citrus plants are rich in endophytic fungi [17]. It is thus possible to find a wide range of active metabolites against Xcc from citrus endophytic fungi. It was found that the secondary metabolites of the EF *Diaporthe* sp. HT-79 isolated from the leaves of a healthy Gannan navel orange tree (*Citrus sinensis* Osbeck cv. Newhall) had significant antibacterial activity against Xcc, and linoleic acid was identified as the most anti-Xcc component, highly inhibiting the Xcc growth via the destruction of the cell membrane and overproduction of reactive oxygen species (ROS) [18]. In our previous study, the citrus endophytic fungus *Nemania* sp. LJZ-Y-11 was found to have significant inhibitory activity against Xcc* in vitro* [19].

*Nemania* species have been reported to possess multiple biological activities, such as antioxidant activity [20], antimalarial activity [21, 22], cytotoxic activity [21–24], cholinesterase inhibitory activity [25], herbicidal activity [22], and antimicrobial activity against animal pathogens [21, 26] as well as fungal plant pathogens [27–29]. However, reports on their antibacterial activity against plant pathogenic bacteria are rare.

In the present study, we aimed to clarify the antibacterial activities of extracts prepared with solvents of different polarities from *Nemania* sp. LJZ-Y-11 against Xcc, isolate and identify the active secondary metabolites, as well as evaluate the effects of the active compound on the Xcc. The results of the present study will encourage further investigation of the secondary metabolites of citrus endophytic fungi and provide a pathway to explore natural control agents of citrus canker.

## Materials and methods

### Reagents and equipment

Beef extract and bacteriological peptone purchased from Guangdong Huankai Microbial Sci. & Tech. Co., Ltd., dextrose purchased from Xilong Science Co., Ltd., and agar powder purchased from Beijing Solarbio Science & Technology Co., Ltd., were used for the preparation of culture media. A copper hydroxide water particle dispersant (Kocide 3000, 46% metallic copper) was purchased from Corteva Agriscience (Shanghai, China), and used as the positive control. Chromatographic reagents petroleum ether (PE), ethyl acetate (EtOAc), *n*-butanol, acetone, dichloromethane, and methanol were of analytical grade, obtained from Xilong Science Co., Ltd, Sichuan, China. Silica gel (100 − 300 mesh, Qingdao Marine Chemical Inc., Qingdao, China) and Sephadex LH-20 (Amersham Biosciences Inc., Shanghai, China) were used for column chromatography. Analytical thin-layer chromatography (TLC) was performed on silica gel GF_254_ plates (Qingdao Marine Chemical Inc., Qingdao, China). The other chemicals, absolute alcohol, NaCl, KCl, Na_2_HPO_4_, and KH_2_PO_4_ obtained from Xilong Science Co., Ltd., and 2.5% glutaraldehyde fixing solution obtained from Shanghai Macklin Biochemical Technology Co., Ltd, were all of analytical grade, used for the preparation of scanning electron microscope samples. Optical rotations were measured at the sodium D line (589 nm) on a WXG-4 disc polarimeter [INESA (Group) Co., Ltd., Shanghai, China]. HRESI-MS/MS spectrum was measured using an Esquire HCT ion trap mass spectrometer (Bruker Daltonics Inc. Billerica, USA) using negative ion mode. NMR spectra were recorded on a Bruker ADVANCE III 500 spectrometer (Bruker, Karlsruhe, Germany). High-performance liquid chromatography (HPLC) analysis was conducted using a Waters e2695-ELS detector (Waters Corporation, Massachusett, USA) with a 4.6 mm × 250 mm i.d. and a 5 μm Agilent Zorbax SB-C_18_ column (Agilent Technologies Inc., California, USA). A Buchi MPLC system (C-615) (BUCHI, Switzerland) with a 250 mm × 10 mm i.d. and a 10 μm COSMOSIL C_18_ column (Nacalai Tesque Inc., Kyoto, Japan) was used for MPLC. Fluorescence microplate read (SpectraMax i3x Platform, Molecular Devices) was used for the determination of the OD value of bacterial suspension. A scanning electron microscope (SEM) (CLARA, Navi, TESCAN) was used for observation of the cell morphology.

### Microbial materials

The pathogen *Xanthomonas citri* subsp. *citri* (Xcc) of citrus canker disease used in this study was provided by the Chemical Ecology Laboratory of the Key Laboratory of Ecology of Rare and Endangered Species and Environmental Protection, Guangxi Normal University. The EF *Nemania* sp. LJZ-Y-11 (GenBank accession number: MK351455) was previously isolated from healthy leaves of *Citrus reticulata* Blanco cv. Shatangju [19].

### Citrus plants

*Citrus reticulata* ‘Orah orange’ provided by Guangxi Institute of Specialty Crops, was used for determining the effects of the active compound isolated from *Nemania* sp. LJZ-Y-11 on the Xcc *in vivo*. The new leaves nearly mature were chosen for the inoculation.

### Culture media

Nutrient agar culture medium (NA) containing 3 g/L of beef extract, 5 g/L of bacteriological peptone, 2.5 g/L of dextrose, and 18 g/L of agar powder, with pH 7.0, was used for the activation culture of Xcc from low-temperature storage tube and the spread plate for the validation of the minimum bactericidal concentration (MBC).

Nutrient in both medium (NB) containing 3 g/L of beef extract, 5 g/L of bacteriological peptone, and 2.5 g/L of dextrose, with pH 7.0, was used for Xcc culture and the preparation of bacterial suspension.

Potato dextrose agar medium (PDA) containing 200 g/L of potato (peeled), 20 g/L of dextrose, 15–20 g/L of agar powder, with pH 7.0, was used for the activation culture of EF *Nemania* sp. LZJ-Y-11.

Potato dextrose broth medium (PDB) containing 200 g/L of potato (peeled), and 20 g/L of dextrose, with pH 7.0, was used for the culture of EF *Nemania* sp. LZJ-Y-11 to prepare the seed liquid.

Rice solid culture medium was prepared as follows: rice and water were 1/1 (m/v), with 100 g of rice per 1000 mL flask. The medium was used for the fermentation culture of EF *Nemania* sp. LZJ-Y-11.

### Fermentation culture of the EF *Nemania* sp. LZJ-Y-11 and the preparation of extracts

According to a previous method with some minor modifications, the EF *Nemania* sp. LZJ-Y-11 was fermented to prepare extracts [19]. The EF *Nemania* sp. LZJ-Y-11 was firstly cultured on the PDA plate at 27 ± 1 °C for 5 days. Then three plugs from the edge of the actively growing colony were inoculated into a 250-mL Erlenmeyer flask containing 100 mL of PDB; the inoculated medium was then incubated on a rotary shaker at 150 rpm and 27 ± 1 °C for 3–5 days. Next, 5 mL of the mycelial suspension was inoculated into a 1000-mL Erlenmeyer flask containing 100 g of rice grain (autoclaved). All flasks were incubated under static conditions, in the dark, at 27 ± 1 °C for 30–45 days. After fermentation, the rice grains, which were covered with profuse fungal growth, were dried and powdered and then were used to prepare an extract using ultrasonication with EtOAc three times. The three filtrates were merged and evaporated to dryness and the crude extract (CE) was obtained. For the preliminary separation, the CE was dissolved in water, and then extracted three times with an equal volume of PE, EtOAc, and *n*-butanol, respectively. The three times filtrates were also merged for each solvent. The remaining water and the merged filtrates were evaporated to dryness to obtain the extracts of water residue, PE, EtOAc, and *n*-butanol, respectively.

### Isolation and identification of compounds from EF *Nemania* sp. LZJ-Y-11

By a bioassay guiding, the EtOAc extract of *Nemania* sp. LZJ-Y-11 was fractionated using silica gel column chromatography with a dichloromethane/methanol mixture (20/1, v/v); we obtained five fractions (Fr. I-V), which were monitored and pooled using TLC analysis. Fr. III was subjected to silica gel chromatography using mixtures of petroleum ether/acetone with increasing polarity (15/1, 10/1, 5/1, 1/1, 0/1, v/v) as eluents to yield 12 subfractions (Frs. 1 to 12). Next, Frs. nine (5 g) was subjected to MPLC (MeOH/H_2_O, 1/5→1/1, v/v) to yield seven subfractions (S1 to S7). S1 (236.0 mg) was purified over a Sephadex LH-20 column (methanol) and chromatographed over silica gel (dichloromethane/methanol, 30/1, v/v) to obtain compound **1** (29 mg). S4 (263.0 mg) was purified over a Sephadex LH-20 column (methanol) and chromatographed over silica gel (dichloromethane/methanol, 45/1, v/v) to obtain compound **2** (20 mg), and S4 (192.0 mg) was purified using HPLC (C_18_, methanol/water, 7/20–4/5, v/v) to obtain compound **3** (18 mg). S5 (142.0 mg) was purified over a Sephadex LH-20 column (methanol) and chromatographed over silica gel (dichloromethane/methanol, 45/1, v/v) to obtain compound **4** (7 mg). S7 (2519.1 mg) was purified over a Sephadex LH-20 column (dichloromethane/methanol, 1/1, v/v) and chromatographed over silica gel (petroleum ether/acetone + 0.1% acid, 8/1→5/1→3/1, v/v) to obtain compound **5** (937.5 mg).

### The culture of *Xanthomonas citri* subsp. *citri* and the preparation of suspension

Xcc preserved at low temperature was firstly inoculated onto an NA plate, and incubated at 27 ± 1℃ for 3 days, repeated transfer once. Then the bacteria were inoculated into a 250-mL Erlenmeyer flask containing 100 mL of NB medium, cultured at 27 ± 1 ℃, 150 rpm for 24 h, to obtain the Xcc suspension liquid (OD value = 0.19, plate count of 10^9^ CFU/mL). The suspension was diluted 100 times to obtain a bacterial suspension with a concentration of 10^7^ CFU/mL and used for the determination of antibacterial activity and the growth curve assay.

### Determination of the antibacterial activity

The antibacterial activity of secondary metabolites from EF *Nemania* sp. LZJ-Y-11 against Xcc was determined as follows. The required concentration of extracts or compounds was dissolved in acetone/water (1/1, v/v) to prepare sample solutions. Then 0.2 mL of the sample solution was evenly mixed with 1.6 mL of NB medium and 0.2 mL of bacterial suspension (prepared as the above method) in a tube, and the acetone/water (1/1, v/v) was used in the same way to prepare a control. Three replicates were set for each treatment and incubated at 27 ± 1℃ in a constant temperature shaking incubator at 150 rpm for 24, 48, and 72 h to observe bacterial growth. The MIC was the minimum concentration at which there was no bacterial growth during 72 h. For the MBC, 50 µL of solutions of the MIC and the higher concentrations were spread on NA plates (in the Petri dish with a diameter of 6 cm), and then incubated at 27 ± 1℃. The growth of Xcc was observed at 24, 48, and 72 h, and the lowest concentration at which there was no growth of bacteria on the NA medium for 72 h was recorded as the MBC.

### The growth curve assay

The effect of the active compound on Xcc growth *in* *vitro* was evaluated by the growth curve assay [30]. The culture of Xcc and the seed suspension were prepared as described in the above method, and the seed suspension (OD600 = 1.9, ≈10^9^ CFU/mL) was diluted 100 times to obtain a bacterial suspension with a concentration of 10^7^ CFU/mL and used for the growth curve assay. This bacterial suspension (2 mL) and NB (16 mL) were added into a 50 mL conical flask, followed by the addition of 2 mL of compound solution (acetone/water, 1/1, v/v, as the solvent, prepared as described in the above method) at series concentrations to achieve a final concentration of 0.03125, 0.06125, 0.125, 0.25, and 0.5 mg.mL^−1^, respectively. The conical flasks were then incubated at 150 rpm and 27 ± 1 °C. Two hundred microliters of incubation solution in the conical flasks were taken out and put into a 96-well plate at the incubation times of 0 h, 8 h, 24 h, 32 h, 48 h, 56 h, and 72 h, respectively. The absorbance of the incubation solution was measured at OD_600_ nm. The mixture of acetone/water (1/1, v/v) prepared in the same way was used as a control. All measurements were performed in triplicate and each experiment contained three replications. The Xcc growth curve was plotted by incubation time versus the OD_600_ values of the incubation solution.

### Scanning electron microscopy (SEM) studies

The effects of the active compound on the Xcc cell morphology were observed by using the SEM [30, 31]. Referring to the above method, Xcc was incubated for 24 h (OD_600_ = 0.19, ≈10^9^ CFU/mL), then 1 mL of the bacterial solution was taken into a 2 mL centrifuge tube and centrifuged at 5000 rpm for 5 min. The supernatant was removed to obtain the bacterial cells, and 0.9 mL of NB medium was added and mixed evenly. The mixture was transferred to a glass tube (1.5 × 10 cm in diameter and length), and 0.1 mL of the sample solution was added to obtain the mixture containing the sample for the final concentrations of 0 (control), 0.03125, 0.0625, 0.125, 0.25, and 0.5 mg.mL^−1^, with 3 replicates for each treatment. After being incubated in a shaking incubator at 27 ± 1℃ and 150 rpm for 10 h, the mixture was centrifuged (5000 rpm, 10 min) to collect the bacterial cells. The bacterial cells were washed twice with PBS solution (pH = 7.0). The bacterial cells were collected by centrifugation, and then, 500 µL glutaraldehyde with a concentration of 2.5% was added and mixed well, fixed at low temperature in the dark at 4℃ for 4 h. Next, the bacterial cells were collected by centrifuging at 5000 rpm for 10 min and washed twice with PBS solution with a pH of 7.0. The bacterial cells were collected by centrifugation. Then dehydrated with 30%, 50%, 70%, 80%, 90%, and anhydrous ethanol in sequence, by standing for 10 min respectively, and the supernatant was removed. An appropriate amount of anhydrous ethanol was added to the bacterial cells and mixed evenly to prepare a bacterial suspension. 1 − 2 µL of the bacterial suspension was dropped onto the mirror surface of the silicon wafer and gold-plated three times, with a duration of 1 min for each time. After the gold-plating was completed, SEM observation was performed.

### Attached leaf assay

The effect of the active compound on Xcc *in vivo* was determined by using an attached leaf assay, referring to Llorens et al. [8], with some modifications, *i.e.*, the Xcc suspension was inoculated into the attached leaves of the citrus plant. The bacterial suspension (0.2 mL, 10^7^ CFU/mL, prepared as the above method) and NB (1.6 mL) were added into a glass tube, followed by the addition of 0.2 mL of compound solution (prepared as described in the above method) to achieve a final concentration of 0.03125, 0.06125, 0.125, 0.25, and 0.5 mg.mL^−1^, respectively. An equal volume of acetone/water (1/1, v/v) was prepared as the control group. Three replicates were set for each group, and all measurements were incubated at 27 ± 1℃ and 150 rpm for 72 h. The culture solution was inoculated into citrus leaves using a previous method with slight modifications [8, 32]. The selected leaves that were newly grown, had reached their maximum leaf area, had just turned green, had grown well, and had not suffered any damage from inoculation. The bacterial solution was injected with a 1-mL sterile needleless syringe on the abaxial leaf blade with approximately 2 µL of bacterial suspension at each injection, and 3 injections were performed on each side of the leaf mid-vein, *i.e.*, a total of 6 injections per leaf for each treatment. Three leaves were inoculated for each sample. Lesion expansion and symptom development were recorded periodically after inoculation, and the photos were taken on the 25th day post-inoculation.

### Statistical analysis

All experiments were performed in triplicate. Statistical analyses, including graphical representations, were performed using Microsoft Excel 2016 (Microsoft Corporation).

## Results

### Antibacterial activities of extracts against *Xanthomonas citri* subsp. *citri*

The antibacterial activities of extracts with different polar solvents from EF *Nemania* sp. LJZ-Y-11 against Xcc are shown in Table 1. Copper hydroxide (the positive control), a common chemical control agent used for Xcc, showed significant antibacterial activities against Xcc *in vitro*, with the MIC and MBC being 0.0625 and 0.125 mg.mL^−1^ at 72 h, respectively. The extracts of EF *Nemania* sp. LJZ-Y-11 displayed varying degrees of antibacterial activity against Xcc, with the MICs and the MBCs ranging from 0.3125 − 5 mg.mL^−1^ and 0.625 − 5 mg.mL^−1^ at 72 h, respectively. Among the tested extracts, the EtOAc extract showed the highest antibacterial activity, with MIC and MBC of 0.3125 and 0.625 mg.mL^−1^, respectively. The results indicated that the active secondary metabolites of EF *Nemania* sp. LJZ-Y-11 against Xcc could be mainly of medium polarity.

**Table 1:** The MICs and MBCs of the extracts with different polar solvents from EF *Nemania* sp. LJZ-Y-11 against Xcc

Extract	MIC (mg.mL^−1^)	MBC (mg.mL^−1^)
PE	5	5
EtOAc	0.3125	0.625
*n*-butanol	2.5	5
water residue	2.5	5
Copper hydroxide	0.0625	0.125

Note: (1) Results for a 72-h culture of Xcc have been presented. (2) PE stands for petroleum ether; EtOAc stands for ethyl acetate. (3) Copper hydroxide, a common chemical control agent used for Xcc, was used as the positive control

### Compounds from the EtOAc extract of endophytic fungus *Nemania* sp. LJZ-Y-11

**Fig. 1:**
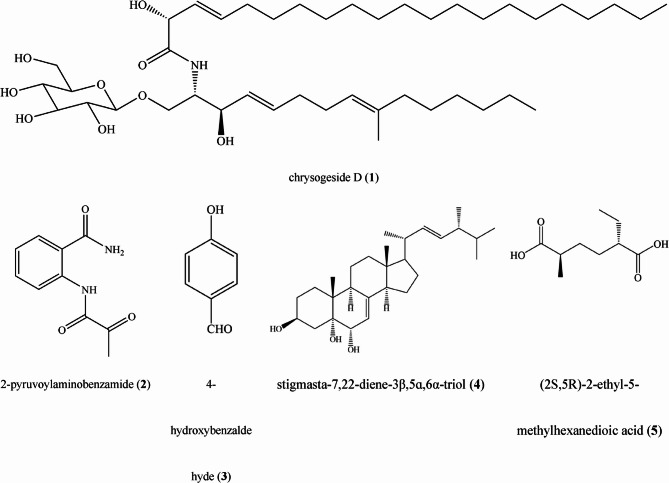
Chemical structures of five compounds isolated from *Nemania* sp. LZJ-Y-11

Five known compounds (**1** − **5**) were isolated from the EtOAc extract of EF *Nemania* sp. LJZ-Y-11. The spectroscopic data are shown in the Supplementary Information. Compound **1** was a white amorphous powder, identified as chrysogeside D by comparative analysis of the spectroscopic data with those in the literature [33]. Compound **2** was a colorless sheet and was identified as 2-pyruvoylaminobenzamide after comparison with reference [34]. Compound **3** was white needle-shaped and identified as 4-hydroxybenzaldehyde, after comparison with reference [35, 36]. Compound **4** was a colorless needle-shaped crystal, which was identified as stigmasta-7,22-diene-3β,5ɑ,6α-triol, after comparison with references [37, 38]. Compound **5** is a white powder, ESI: m/z [M-H]^−^ =187.0972, [α]^25^_D_ + 4.5° (*c* 0.1, MeOH), after comparison with reference [39], was identified as (2*S*,5*R*)−2-ethyl-5-methylhexanedioic acid. The chemical structures of five compounds are shown in Fig. 1.

### Antibacterial activities of the five compounds against *Xanthomonas citri* subsp. *citri*

The antibacterial activity of the above five compounds against Xcc was determined (Table 2). At a tested concentration of 0.25 mg.mL^−1^, both compound **3** and compound **5** exhibited inhibitory activity against Xcc during 72 h, while the other three compounds did not show antibacterial activity. The MIC and MBC of compound **5** against Xcc were 0.125 and 0.25 mg.mL^−1^, respectively. The MIC of compound **3** was 0.25 mg.mL^−1^, with MBC > 0.25 mg.mL^−1^. The results indicated that compound **5** exhibited superior antibacterial activity against Xcc. In addition, the antibacterial activity of the compound **5** against Xcc is close to that of the positive control copper hydroxide, a common chemical control agent used for Xcc (MIC 0.0625 mg.mL^−1^, MBC 0.125 mg.mL^−1^) (Table 2).

**Table 2:** Inhibitory effects of the five active compounds isolated from the EtOAc extract of *Nemania* sp. LJZ-Y-11 against Xcc

Compound	Inhibition (0.25 mg.mL^−1^)	MIC (mg.mL^−1^)	MBC (mg.mL^−1^)
**1**	+++	> 0.25	> 0.25
**2**	+++	> 0.25	> 0.25
**3**	−−−	0.25	> 0.25
**4**	+++	> 0.25	> 0.25
**5**	−−−	0.125	0.25
Copper hydroxide	−−−	0.0625	0.125

Note: (1) Results for a 72-h culture of Xcc have been presented. (2) “−” indicates no bacterial growth, and “+” indicates bacterial growth. (3) Copper hydroxide, a common chemical control agent used for Xcc, was used as the positive control

### The effect of compound 5 on the growth of *Xanthomonas citri* subsp. *citri in vitro*

**Fig. 2:**
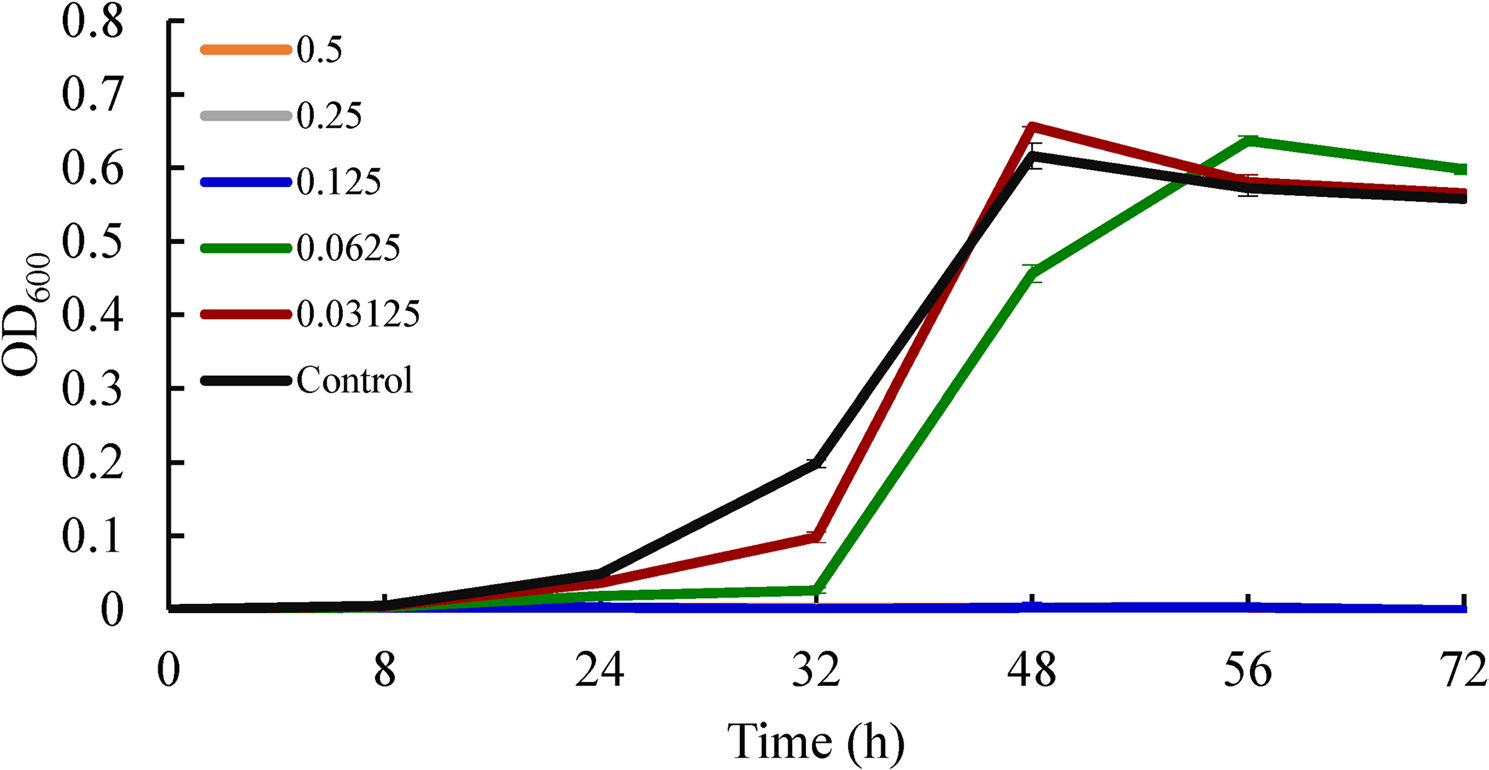
Effects of compound **5** on the growth of *Xanthomonas citri* subsp. *citri* [*Xanthomonas citri* subsp. *citri* (Xcc) was treated by compound **5** with a series of concentrations to achieve a final concentration of 0.03125, 0.06125, 0.125, 0.25, and 0.5 mg.mL^−1^, respectively. The mixture of acetone/water (1/1, v/v) prepared in the same way was used as a control. The absorbance of the incubation solution was measured at OD_600_ nm at the incubation times of 0 h, 8 h, 24 h, 32 h, 48 h, 56 h, and 72 h, respectively. The Xcc growth curve was plotted by incubation time versus the OD_600_ values of the incubation solution]

As shown in Fig. 2, the inhibitory effect of compound **5** on the Xcc growth displayed a clear dose-dependent pattern. Compared with the blank control, compound **5** entirely inhibited the growth of Xcc at the treated concentrations of 0.125 mg.mL^−1^ (MIC), 0.25 mg.mL^−1^ (MBC), and 0.5 mg.mL^−1^ (2MBC). Even when treated with low concentrations of 0.03125 and 0.0625 mg.mL^−1^, the logarithmic phase of Xcc was delayed, though the OD_600_ value for the cells in the stationary phase had not significantly decreased as well in comparison to the blank control.

### Effects of compound 5 on the cell morphology of *Xanthomonas* *citri* subsp. *citri*

**Fig. 3:**
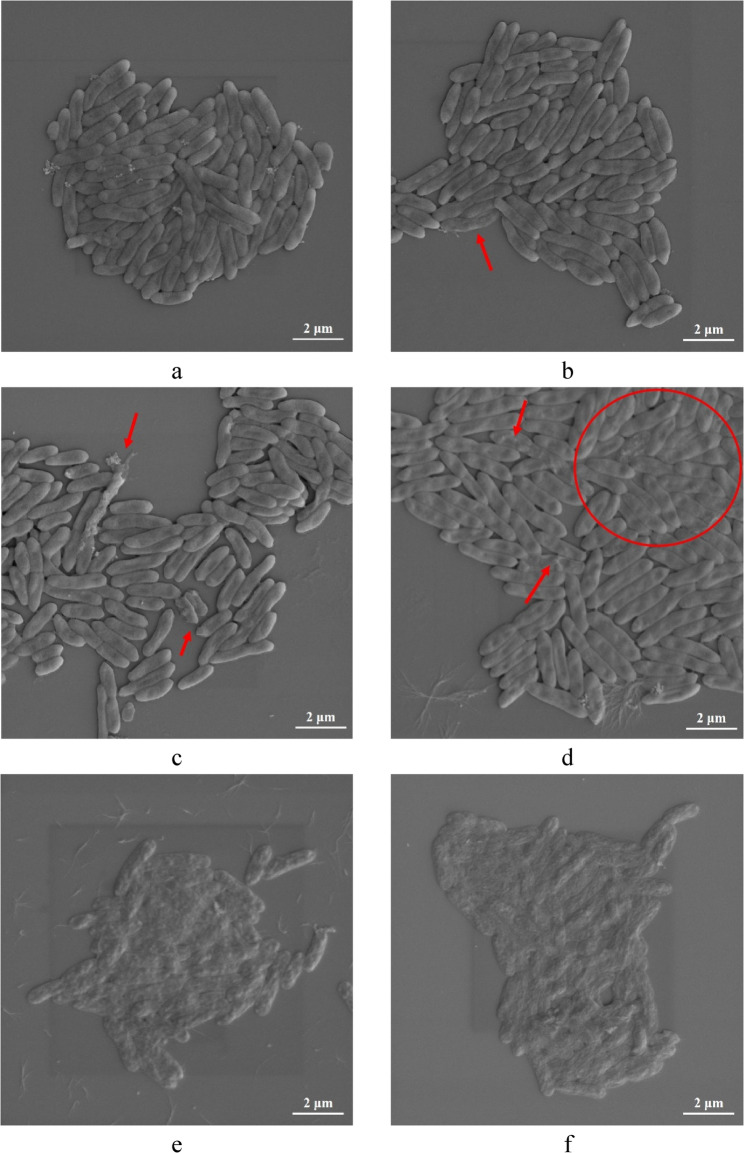
Effects of compound **5** on the cell morphology of *Xanthomonas citri* subsp. *citri* [The morphological changes of *Xanthomonas citri* subsp. *citri* (Xcc) cells were observed by SEM; (**a**) was the control group that treated Xcc cells with acetone/water (1/1, v/v) (the solvent of compound **5**); (**b**)−(**f**) were the treated groups in which Xcc cells were treated with concentrations of 0.03125, 0.0625, 0.125, 0.25, and 0.5 mg.mL^−1^ of compound **5** for 10 h, respectively.]

To elucidate the growth inhibitory effect of compound **5** on Xcc, the effects of different concentrations of compound **5** on the Xcc cell morphology were investigated by SEM (Fig. 3). The results indicated that the influence of compound **5** on Xcc cell morphology was also in a dose-dependent manner. Compound **5** destroyed almost all cells at the concentrations of 0.25 mg.mL^−1^ (MBC) and 0.5 mg.mL^−1^ (2MBC) (Fig. 3e, f). When the cells were treated with the concentration of 0.125 mg.mL^−1^ (MIC), some of the Xcc cells became flattened and shriveled, with rough surfaces (Fig. 3d). With the decreasing of the concentration to 0.03125 and 0.0625 mg.mL^−1^, a very small number of cells were affected with rough surfaces or rupture (Fig. 3b, c). Interestingly, except the bactericidal concentrations (0.25 and 0.5 mg.mL^−1^) destroyed the cells and made them crumpled together into clusters (Fig. 3e, f), under the other three concentrations (0.03125, 0.0625, and 0.125 mg.mL^−1^) treatment (Fig. 3b − d), the cells were significantly dispersed compared to the control group (Fig. 3a), suggesting that it might be related to the inhibition of biofilm formation.

### The effect of compound 5 on the growth of *Xanthomonas citri* subsp.* citriin vivo*

**Fig. 4:**
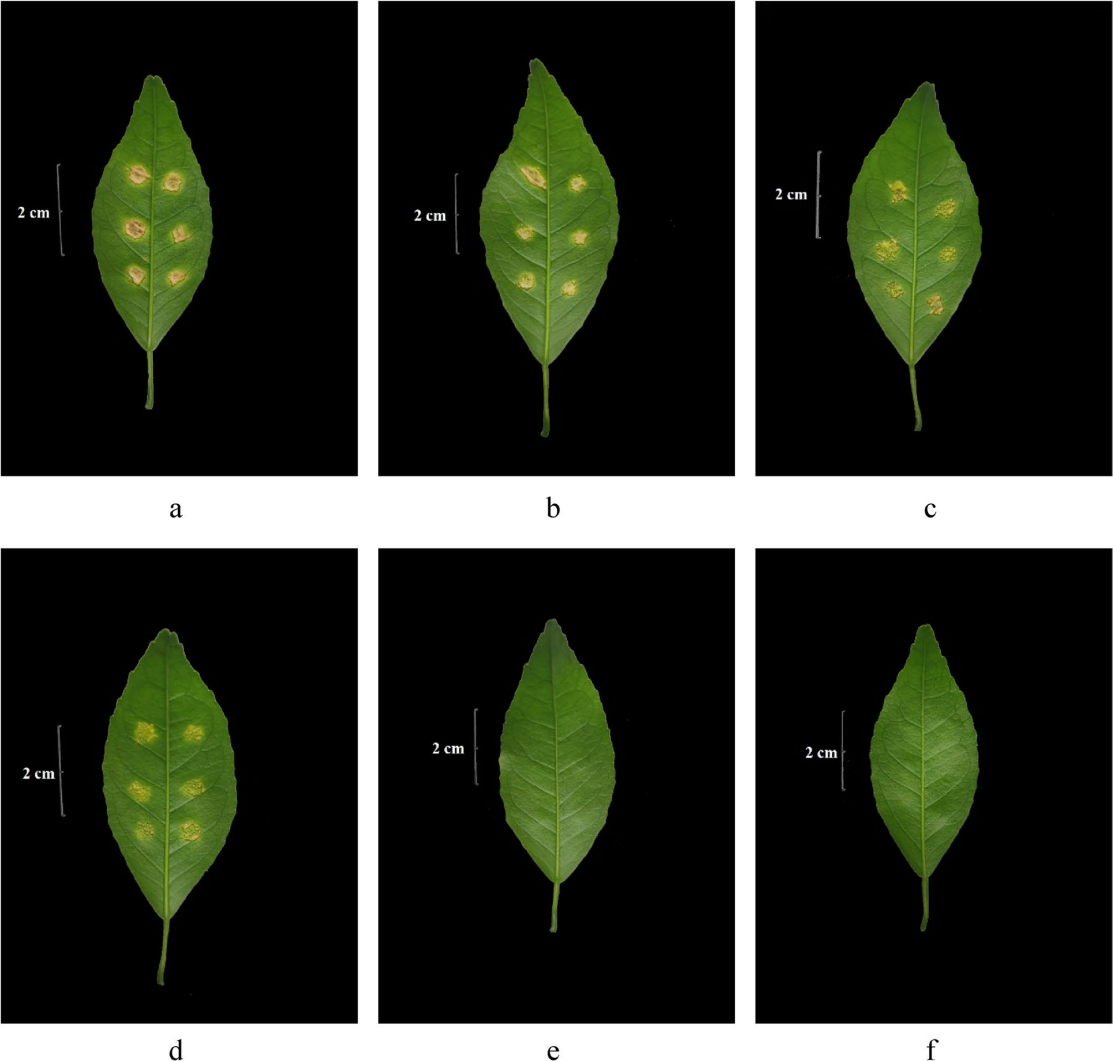
The formation of citrus canker lesions in the attached citrus leaves after inoculation with active compound-treated and untreated *Xanthomonas citri* subsp. *citri* [The pictures of a − f were taken on the 25th day after inoculation; (**a**) was the control group that treated *Xanthomonas citri* subsp. *citri* (Xcc) cells with acetone/water (1/1, v/v) (the solvent of compound **5**); (**b**)−(**f**) were the treated groups in which Xcc cells were treated with concentrations of 0.03125, 0.0625, 0.125, 0.25, and 0.5 mg.mL^−1^ of compound **5** for 72 h, respectively.]

To further clarify the effect of compound **5** on the pathogenicity of Xcc, we observed the formation of lesions of the attached citrus leaves after inoculation of compound-treated Xcc (Fig. 4). Xcc treated with concentrations of 0.03125 mg.mL^−1^, 0.0625 mg.mL^−1^, and 0.125 mg.mL^−1^ could all cause canker lesions in citrus leaves (Fig. 4b-d). However, the degree of suberization of the lesions was lower than that in the control groups (Fig. 4a), especially in the treatments with concentrations of 0.0625 mg.mL^−1^ and 0.125 mg.mL^−1^ (MIC) (Fig. 4c, d), where a single lesions spot could be seen at each inoculation point instead of the lesions spots on each inoculation point turned into a whole piece with suberization in the control group. In addition, the pathogenic bacteria Xcc treated with MBC (0.25 mg.mL^−1^) and 2MBC (0.5 mg.mL^−1^) did not form any lesions in the inoculated leaves. The results indicated that the effects of active compound **5** on the formation of lesions of Xcc in citrus leaves were dose-dependent.

**Fig. 5:**
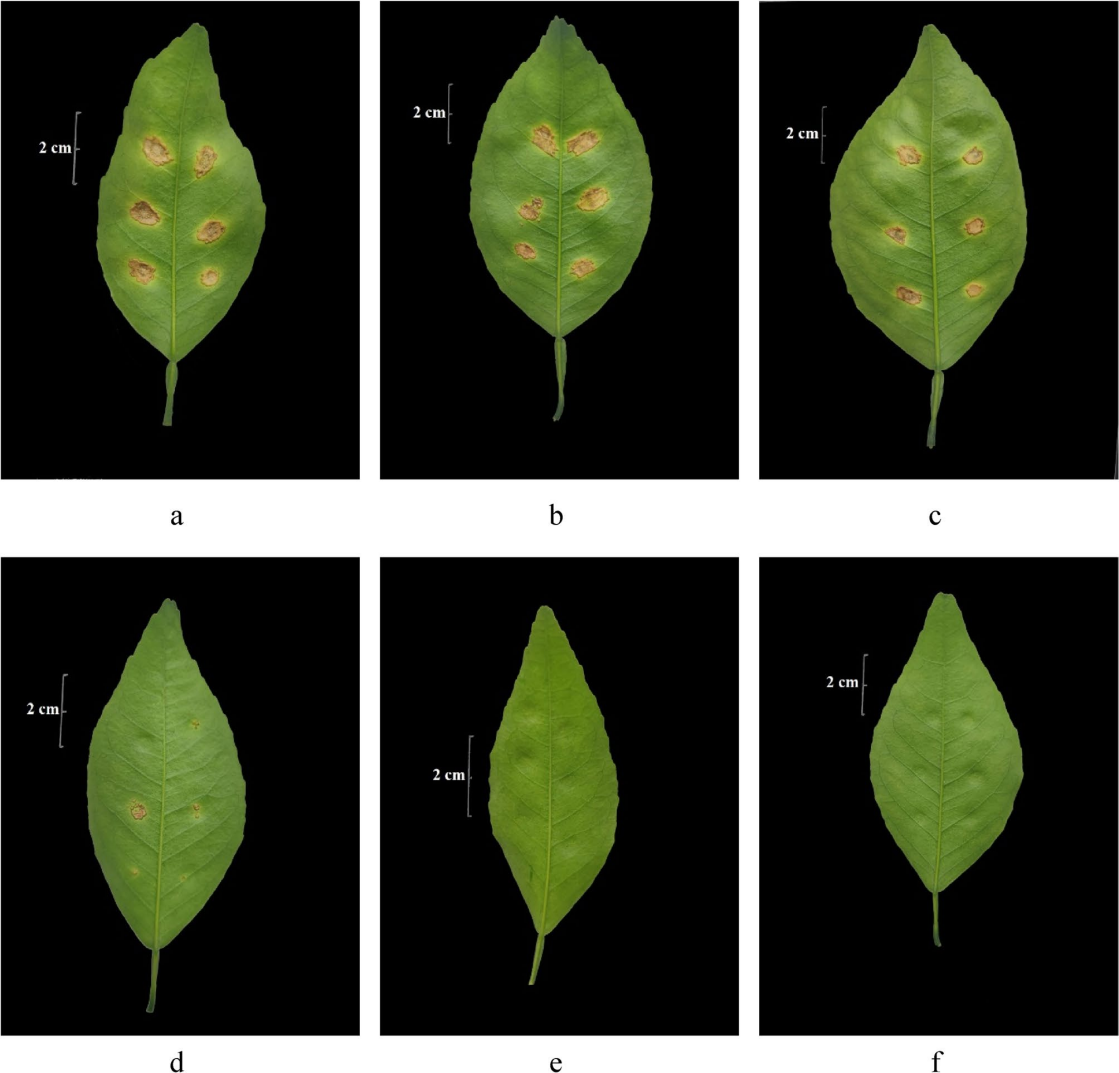
The formation of citrus canker lesions in the attached citrus leaves after inoculation with active EtOAc extract-treated and untreated *Xanthomonas citri* subsp. *citri* [The pictures of a − f were taken on the 25th day after inoculation; (**a**) was the control group that treated *Xanthomonas citri* subsp. *citri* (Xcc) cells with acetone/water (1/1, v/v) (the solvent of EtOAc extract); (**b**)−(**f**) were the treated groups in which Xcc cells were treated with concentrations of 0.125, 0.25, 0.3125, 0.625, and 1.25 mg.mL^−1^ of EtOAc extract for 72 h, respectively.]

**Fig. 6:**
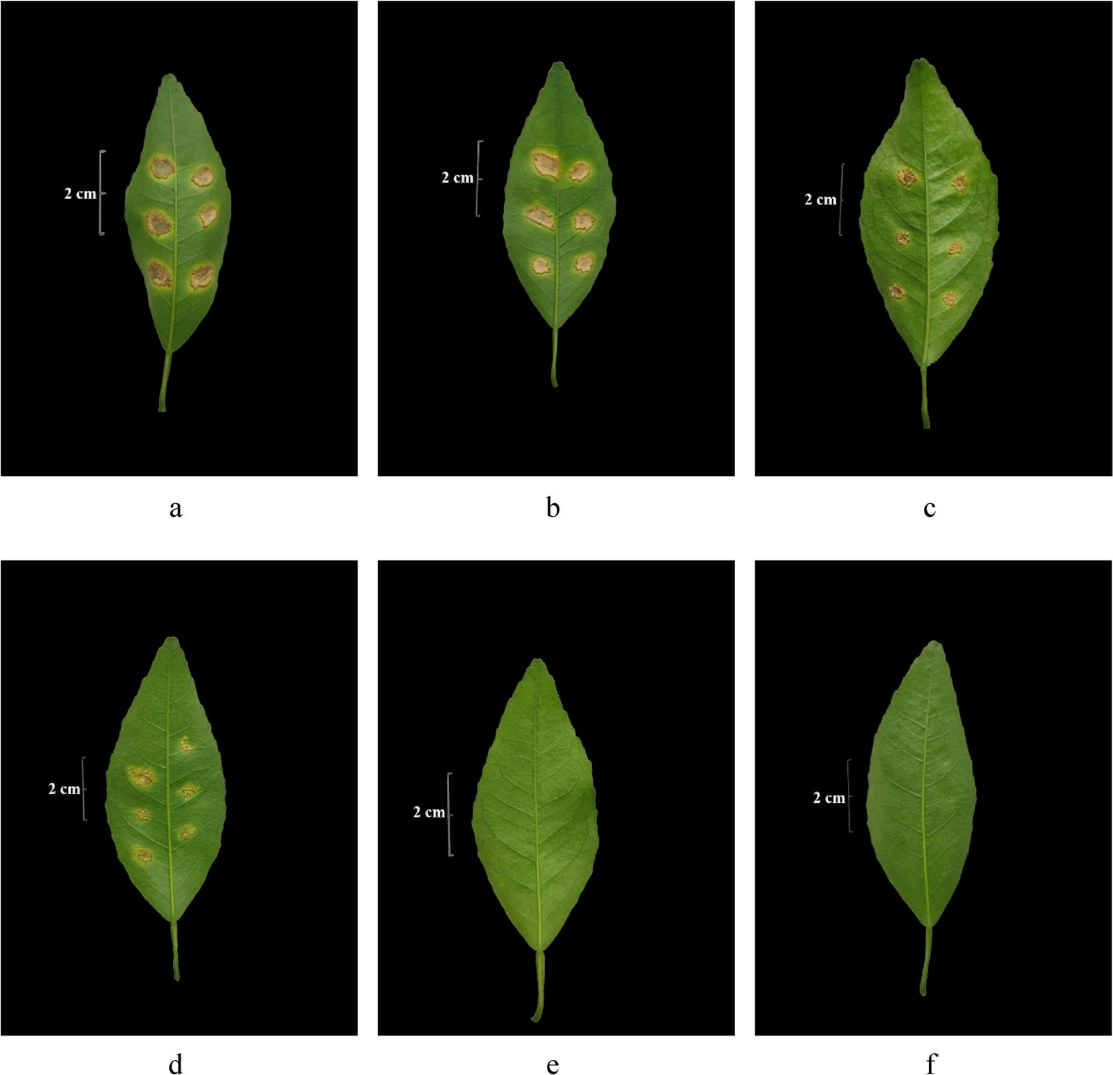
The formation of citrus canker lesions in the attached citrus leaves after inoculation with copper hydroxide-treated and untreated *Xanthomonas citri* subsp. *citri* [The pictures of a − f were taken on the 25th day after inoculation; (**a**) was the control group that treated *Xanthomonas citri* subsp. *citri* (Xcc) cells with water (the solvent of copper hydroxide); (**b**)−(**f**) were the treated groups in which Xcc cells were treated with concentrations of 0.03125, 0.05, 0.0625, 0.125, and 0.25 mg.mL^−1^ of copper hydroxide for 72 h, respectively.]

Similar results were also found in EtOAc extract-treated Xcc (Fig. 5) and copper hydroxide-treated Xcc (Fig. 6). Similarly, no lesions could be forming on the citrus leaves when the Xcc was treated by the MBC or much higher concentrations of EtOAc extract (MBC 0.625 mg.mL^−1^, 2MBC 1.25 mg.mL^−1^) or copper hydroxide (MBC 0.125 mg.mL^−1^, 2MBC 0.25 mg.mL^−1^), and a few lesions shown on the citrus leaves with the Xcc treated by the MIC or the next concentration of EtOAc (MIC 0.3125 mg.mL^−1^) or copper hydroxide (MIC 0.0625 mg.mL^−1^).

## Discussion

Among the extracts prepared with solvents of different polarities from EF *Nemania* sp. LJZ-Y-11, EtOAc extract showed better antibacterial activities against citrus canker pathogen Xcc with MIC and MBC respectively at 0.3125 and 0.625 mg.mL^−1^, which was higher than some extracts from plants or microorganisms reported in literature, for example, the essential oils from *Citrus aurantium* and *C. aurantifolia* and their constituents (MBC = 0.725–14.5 mg.mL^−1^) [9], EtOAc extract and butanol extract of pomelo (*Citrus grandis* Osbeck) seeds (MIC at 2.5 and 5 mg.mL^−1^, respectively) [40]; as well as the extracts from terrestrial and marine Antarctic fungi *Pseudogymnoascus* sp. and *Penicillium* sp. (MBC at 1.5–2.1 mg.mL^−1^) [41].

Five compounds were obtained from the EtOAc extract of *Nemania* sp. LJZ-Y-11, and compounds 4-hydroxybenzaldehyde (**3**) and (2*S*,5*R*)−2-ethyl-5-methylhexanedioic acid (**5**) displayed better antibacterial activities, especially (2*S*,5*R*)−2-ethyl-5-methylhexanedioic acid (**5**) showed the best antibacterial activity against Xcc with MIC of 0.125 mg.mL^−1^ and MBC of 0.25 mg.mL^−1^, closing to that of the positive control copper hydroxide (MIC 0.0625 mg.mL^−1^, MBC 0.125 mg.mL^−1^), a commonly chemical control agent used for Xcc. The antibacterial potential of compound **5** was also comparable or superior to some natural products and synthetic agents, for example, higher than the constituents of the essential oils of *Citrus aurantium* and *C. aurantifolia* (MICs 0.375 − 8.5 mg.mL^−1^; MBCs 0.725–14.5 mg.mL^−1^) [9], as well as the commercial active compounds bismerthiazol and thiodiazole copper (EC_50_ 0.068 mg.mL^−1^ and 0.057 mg.mL^−1^) [12]; and comparable to the recombinant peptide CAP18 (MIC of 0.080 mg.mL^−1^) [11], as well as some of the components (MIC of 0.0078 − 0.25 mg.mL^−1^) identified in the EF *Diaporthe* sp. HT-79 which was isolated from the leaves of a healthy Gannan navel orange tree (*Citrus sinensis* Osbeck cv. Newhall) [18]. The above results indicated that (2*S*,5*R*)−2-ethyl-5-methylhexanedioic acid (**5**) was an important active substance in the citrus endophytic fungus *Nemania* sp. LJZ-Y-11.

The effects of active compound **5** on the growth and cell morphology of Xcc were dose-dependent. The low concentration (0.03125 mg.mL^−1^, *i.e.*, 1/4 MIC) of compound **5** showed less impact on the growth of Xcc, and the bacteria numbers in the stationary phase had not significantly decreased as well in comparison to the blank control, which was similar to the effect of 1/32 MIC concentration of actinomycin X_2_ on Xcc [42]. The treatment with a concentration of 0.0625 mg.mL^−1^ could delay the time of Xcc to reach the logarithmic phase, though not affect the bacteria numbers in the stationary phase compared with the blank control. In addition, the treatment with 0.125 mg.mL^−1^ (MIC), 0.25 mg.mL^−1^ (MBC), and higher concentration could all completely inhibit the growth of Xcc. The results were consistent with the effects of compound **5** on the cell morphology, *i.e.*, the destructive effects on cell morphology increase with the increment of the treated concentration. The treatment of 0.125 mg.mL^−1^ (MIC) significantly increased the morphological changes of Xcc cells, indicating that it not only inhibited cell proliferation but also had a destructive effect on cells. Moreover, the treatment of MBC (0.25 mg.mL^−1^) as well as higher concentration (0.5 mg.mL^−1^, 2MBC) destroyed almost all the Xcc cells, leading to the death of cells, which is consistent with the findings of the growth curve measurement. The results indicated that compound **5** had a good anti-Xcc potential.

Compound **5** could also affect the growth of Xcc in leaves and the formation of lesions, resulting in fewer lesions (treated with 0.03125 − 0.125 mg.mL^−1^) or no lesions formed (treated with MBC and 2MBC) in the leaves. Combining the results of growth curve analysis and cell morphology assay, it was found that although the treatment of 0.03125 mg.mL^−1^ and 0.0625 mg.mL^−1^ had little effect on the growth and cell morphology of Xcc, the treatments might inhibit the activity of related virulence factors [43], thereby reducing the pathogenicity of Xcc in leaves. It was reported that the lipopeptide extract of *Bacillus amyloliquefaciens* F9 effectively inhibited the growth and extracellular enzyme activity of Xcc jx-6 [43]. The specific mechanism of action could be explored in the later stage. It was worth mentioning that the MBC and higher concentration led to cell death by disrupting cell structure and completely prevented lesions formed in citrus leaves. In further study, field control experiments could be conducted to provide a reference for the application of the active compound of EF *Nemania* sp. LJZ-Y-11 in the prevention and control of citrus canker disease.

Reports on the antibacterial activity of *Nemania* secondary metabolites against plant pathogenic bacteria were rare, though some bioactive compounds had already been isolated from *Nemania* species [21–25]. To our knowledge, it is the first report of the **5** compounds isolated from *Nemania* species. Among the five compounds, (2*S*,5*R*)−2-ethyl-5-methylhexanedioic acid (**5**) was first isolated as a hexanedioic acid analogue from the endophytic fungus of *Orchidantha chinensis*, *Penicillium* sp. OC-4, and was reported to have strong antioxidant activity against superoxide anion radicals as well as a low antibacterial activity against animal pathogenic bacteria [39]. Additionally, hexanedioic acid has been reported to possess antibacterial properties that effectively inhibit the growth/proliferation of pathogenic bacteria, including methicillin-resistant *Staphylococcus aureus* (MRSA) [44]. These studies suggest the utilization of hexanedioic acid as well as its analogues in the antibacterial field. The active compound 4-hydroxybenzaldehyde (**3**) is distributed in medicinal plants, possesses multiple physiological functions [45], and is an important organic intermediate that is widely used in medicine, pesticides, food flavoring, the cosmetics industry, and other applications. The compound 4-hydroxybenzaldehyde was also isolated from many fungal species, such as the endophytic fungus of *Aquilaria sinensis* (Lour.) Gilg, *Phaeoacremonium rubrigenum* [46]; the endophytic fungus of *Cordyceps sinensis*, *Penicillium herquei* [47]; and the endophytic fungus of *Lycopersicon esculentum*, *Acremonium implicatum* [48], and was found to have significant antibacterial, antifungal, antioxidant, and nematode activities. In addition, 4-hydroxybenzaldehyde (**3**) had a remarkable antibacterial inhibitory effect (IC_50_ of 14.1 µg/mL) against the phytopathogen *Xanthomonas vesicatoria* [48]. The above research indicated that 4-hydroxybenzaldehyde (**3**) and (2*S*,5*R*)−2-ethyl-5-methylhexanedioic acid (**5**) were important active substances and have great potential for application in the control of citrus canker.

## Conclusion

This study found that endophytic fungi in citrus could produce active products against the citrus canker pathogen, and both crude extracts and compounds of EF *Nemania* sp. LJZ-Y-11 had good antibacterial activity. The research results provided an experimental basis for their future application in the control of citrus canker and indicated that there is great potential to find bioactive substances against Xcc from citrus endophytic fungi. While the structures of the five compounds need to be further investigated, as well as their other bioactivities could also be studied. In addition, further exploration of the active metabolites of EF *Nemania* sp. LJZ-Y-11 as well as the other endophytic fungi could be conducted in the future, and field experiments could also be carried out to promote the application of secondary metabolites of citrus endophytic fungi in the prevention and control of citrus canker disease.

## Supplementary Information

Supplementary Material 1

## Data Availability

Data is provided within the manuscript or supplementary information files.
