# Supplementary material for: The effects of the secondary metabolites of the citrus endophytic fungus Nemania sp. LJZ-Y-11 on the citrus canker disease-causing pathogen Xanthomonas citri subsp. citri
Source: BMC Microbiol. 2025 Oct 21;25:670. doi: 10.1186/s12866-025-04403-8 (PMC12538939; doi:10.1186/s12866-025-04403-8)
Supplement: Supplementary file 1 — Supplementary Material 1 [file 12866_2025_4403_MOESM1_ESM.pdf]

Supporting Information

The effects of the secondary metabolites of the citrus endophytic fungus *Nemania* sp. LJZ-Y-11 on the citrus canker disease-causing pathogen *Xanthomonas citri* subsp. *citri*

Xueyu Chen[a, b, c, #], Qicong Li[a, b, c, #], Jie Yao[a, b, c], Linfang Huang[a, b, c], Rourou Lu[a, b, c], Zhiyong Deng[a, b, c], Haiyu Luo[a, b, c, *], Yecheng Deng[a, b, c, *], Xianglin Xu[d, *]

[a] *Key Laboratory of Ecology of Rare and Endangered Species and Environmental Protection, Guangxi Normal University, Ministry of Education, Guilin Guangxi 541006, China*

[b] *Guangxi Key Laboratory of Landscape Resources Conservation and Sustainable Utilization in Lijiang River Basin, Guangxi Normal University, Guilin Guangxi 541006, China*

[c] *Guangxi Key Laboratory of Rare and Endangered Animal Ecology, Guangxi Normal University, Guilin Guangxi 541006, China*

[d] *School of food and health, Guilin Tourism University, Guilin Guangxi 541006, China*

#These authors contributed to the work equally and should be regarded as co-first authors

List of Supporting Information

The spectroscopic data of five compounds 1

Figure S1 The ^{1}H NMR spectrum of chrysogeside D (**1**) (600 MHz, MeOD) 3

Figure S2 The ^{13}C NMR spectrum of chrysogeside D (**1**) (600 MHz, MeOD) 4

Figure S3 The ^{1}H NMR spectrum of 2-pyruvoylaminobenzamide (**2**) (400 MHz, DMSO-d_6). 5

Figure S4 The ^{13}C NMR spectrum of 2-pyruvoylaminobenzamide (**2**) (100 MHz, DMSO-d_6) 6

Figure S5 The ^{1}H NMR spectrum of 4-hydroxybenzaldehyde (**3**) (400 MHz, MeOD) 7

Figure S6 The ^{13}C NMR spectrum of 4-hydroxybenzaldehyde (**3**) (100 MHz, MeOD) 8

Figure S7 The ^{1}H NMR spectrum of stigmasta-7,22-diene-3β,5α,6α-triol (**4**) (400 MHz, DMSO-d_6) 9

Figure S8 The ^{13}C NMR spectrum of stigmasta-7,22-diene-3β,5α,6α-triol (**4**) (100 MHz, DMSO-d_6) 10

Figure S9 The ^{1}H NMR spectrum of (2*S*,5*R*)-2-ethyl-5-methylhexanedioic acid (**5**) (600 MHz, MeOD) 11

Figure S10 The ^{13}C NMR spectrum of (2*S*,5*R*)-2-ethyl-5-methylhexanedioic acid (**5**) (150 MHz, MeOD) 12

Figure S11 The ESIMS/MS of (2*S*,5*R*)-2-ethyl-5-methylhexanedioic acid (**5**) 13

Figure S12 The HPLC chromatogram of (2*S*,5*R*)-2-ethyl-5-methylhexanedioic acid (**5**) 14

The spectroscopic data of five compounds

Chrysogeside D (**1**): ^{1}H-NMR (600 MHz, MeOD): δ 5.68 (1H, dt, *J* = 15.4, 6.4 Hz, H-4′), 5.56 (1H, dt, *J* = 15.4, 6.6 Hz, H-5), 5.43 (1H, dd, *J* = 15.4, 4.4 Hz, H-3′), 5.36 (1H, dd, *J* = 15.4, 6.6 Hz, H-4), 5.09 (1H, br t, *J* = 6.6 Hz, H-8), 4.30 (1H, br t, *J* = 5.5 Hz, H-2′), 4.10 (1H, d, *J* = 7.7 Hz, H-1′′), 3.97 (1H, m, H-3), 3.95 (2H, m, H-1), 3.78 (1H, m, H-2), 3.66 (2H, br dd, *J* = 9.9, 6.6 Hz, H-6′′), 3.50 (2H, dd, *J* = 11.0, 4.4 Hz, H-1), 3.43 (2H, ddd, *J* = 12.1, 6.6, 5.5 Hz, H-6′′), 3.13 (1H, m, H-3″), 3.08 (1H, m, H-5′′), 3.04 (1H, m, H-4″), 2.95 (1H, m, H-2′′), 1.93 (2H, m, H-6,7,10,5′), 1.54 (3H, br s, H-CH_3-9), 1.32 (2H, m, H-16,18′), 1.29 (2H, m, H-12,15,17′), 1.23 (2H, m, H-11,13,14,6′–16′), 0.85 (3H, t, *J* = 6.6 Hz, H-17,19′). ^{13}C-NMR (150 MHz, MeOD): δ 29.2×10, 135.1, 131.2, 131.2, 131.2, 129.1, 123.7, 103.6, 77.0, 76.6, 73.5, 72.1, 70.7, 70.2, 68.8, 61.2, 53.0, 39.5, 31.8, 31.8, 31.5, 31.5, 29.2, 29.2, 28.9, 28.9, 27.5, 27.5, 22.3, 22.3, 15.9, 172.3, 14.1, 14.1.

2-pyruvoylaminobenzamide (**2**): ^{1}H NMR (400 MHz, DMSO-d_6): δ 12.66, 8.31, 7.77 (each 1H, br s, NH), 8.59 (1H, dd, *J* = 8.2, 1.2 Hz, 3-H), 7.85 (1H, dd, *J* = 8.2, 1.2 Hz, 6-H),7.57 (1H, td, *J* = 8.2, 1.2 Hz, 4-H), 7.21 (1H, td, *J* = 8.2, 1.2 Hz, 5-H), 5.75 (s, 1H), 2.43 (3H, s, 10-H_3). ^{13}C NMR (100 MHz, DMSO-d_6): δ 196.9, 170.8, 159.2, 138.6, 132.8, 129.8, 123.9, 121.2, 120.3, 24.6.

4-hydroxybenzaldehyde (**3**): ^{1}H NMR (400 MHz, MeOD): δ 9.78 (s, -CHO),7.79 (2H, d, J = 8.6 Hz, H-2, 6), 6.93 (2H, d, J = 8.6 Hz, H-3, 5). ^{13}C NMR (100 MHz, MeOD): δ 191.5, 163.8, 132.1, 128.9, 115.5.

Stigmasta-7,22-diene-3β,5α,6α-triol (**4**): ^{1}H NMR (400 MHz, DMSO-d_6): δ 5.23 (1H, dd, *J* = 6.9, 15.3 Hz, 22-H), 5.22 (1H, dd, *J* = 7.8, 15.3 Hz, 23-H), 5.09 (1H, s, 7-H), 3.77 (1H, m, 3-H), 3.17 (1H, s, 6-H), 1.85–1.98 (6H, m, H-2, H-9, H-12, H-20, H-24), 1.72-1.83 (1H, m, 14-H), 1.62–1.69 (2H, m, 16-H), 1.22–1.52 (11H, m, H-1, H-2, H-4, H-11, H-15, H-17, H-25), 1.

00 (3H, d, *J* = 6.6 Hz, 21-H), 0.91 (3H, s, 19-H), 0.88 (3H, d, *J* = 6.8 Hz, 27-H), 0.81 (3H, d, *J* = 5. 3 Hz, 28-H), 0.79 (3H, d, *J* = 6.8 Hz, 26-H), 0.55 (3H, s, 18-H). ^{13}C NMR (100 MHz, DMSO-d_6): δ 139.7, 135.4, 131.4, 119.4, 74.4, 72.2, 65.9, 55.3, 54.2, 43.0, 42.2, 42.0, 40.2, 40.0, 39.0, 36.6, 32.4, 31.2, 27.7, 22.6, 21.3, 21.0, 19.7, 19.4, 17.7, 17.3, 12.0.

(2*S*,5*R*)-2-ethyl-5-methylhexanedioic acid (**5**): ^{1}H NMR (600 MHz, MeOD): δ 2.56 (m, 2H), 1.61 (m, 1H), 1.49 (m, 1H), 1.39–1.25 (overlapped, 4H), 1.16 (d, *J* = 6.5 Hz, 3H), 0.90 (t, *J* = 6.8 Hz, 3H). ^{13}C NMR (150 MHz, MeOD): δ 178.8, 178.1, 50.2, 43.5, 31.7, 30.7, 23.5, 16.0, 14.2. ESI-MS *m/z* $[M – H]^{-}$=187.0972. $[\alpha]^{25}_{D}$ + 4.5 (*c* 0.1, MeOH).

Figure S1 The ^{1}H NMR spectrum of chrysogeside D (**1**) (600 MHz, MeOD)

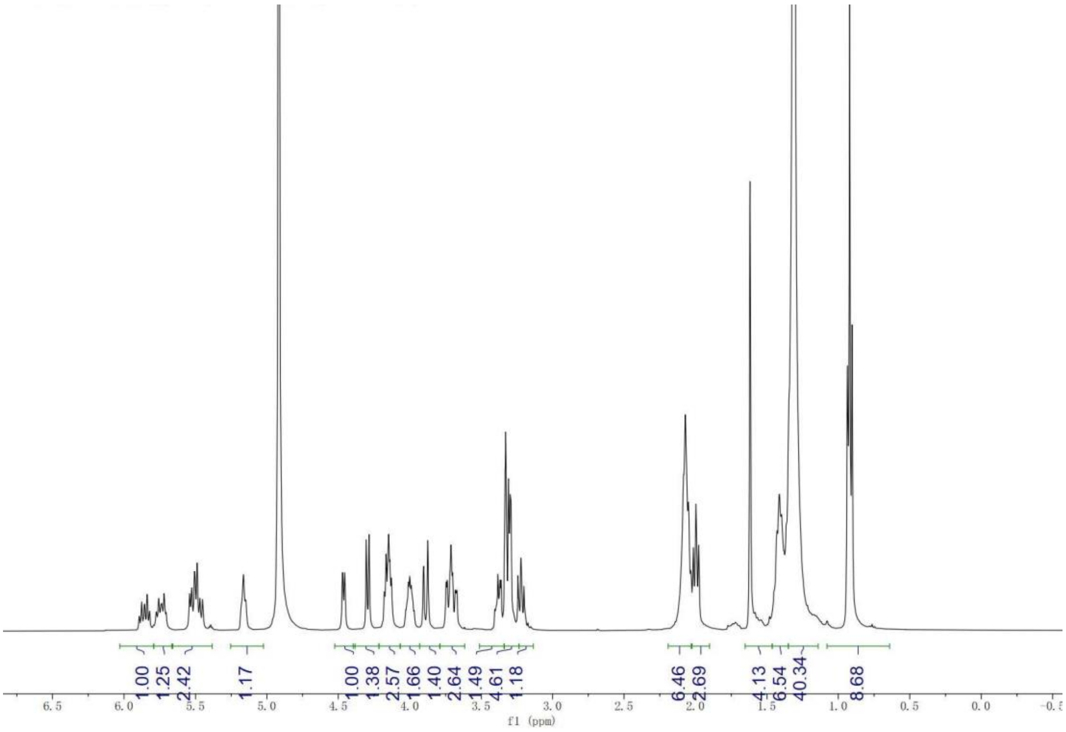

Figure S2 The ^{13}C NMR spectrum of chrysogeside D (**1**) (600 MHz, MeOD)

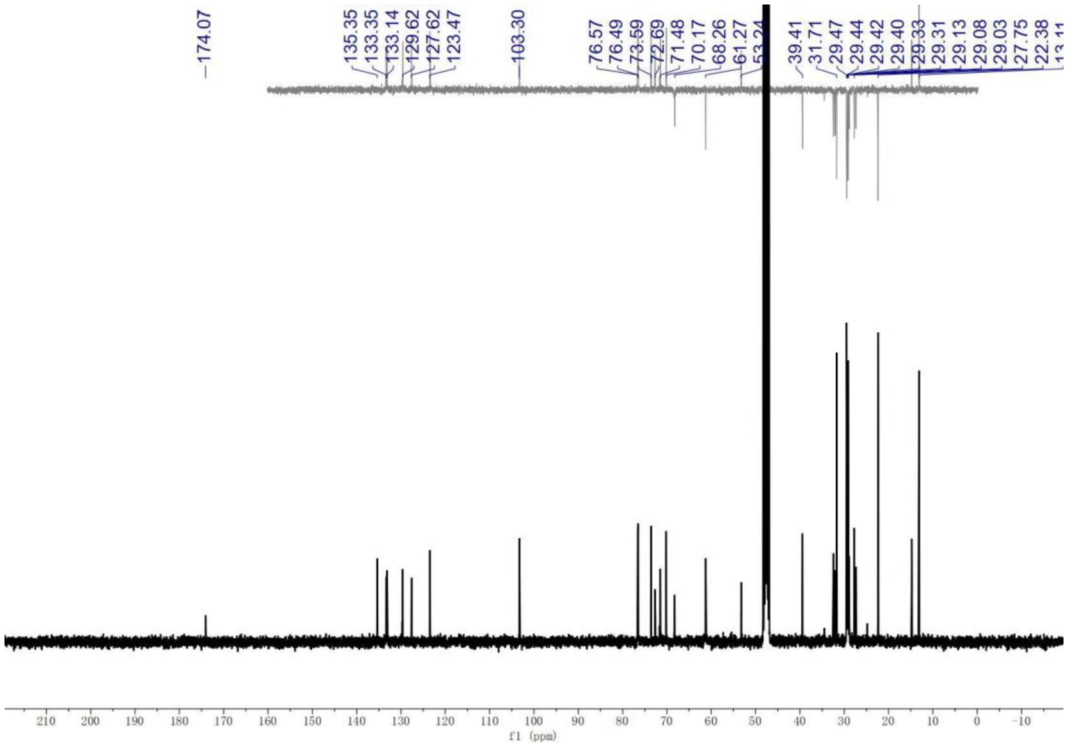

Figure S3 The ^{1}H NMR spectrum of 2-pyruvoylaminobenzamide (**2**) (400 MHz, DMSO-d_6)

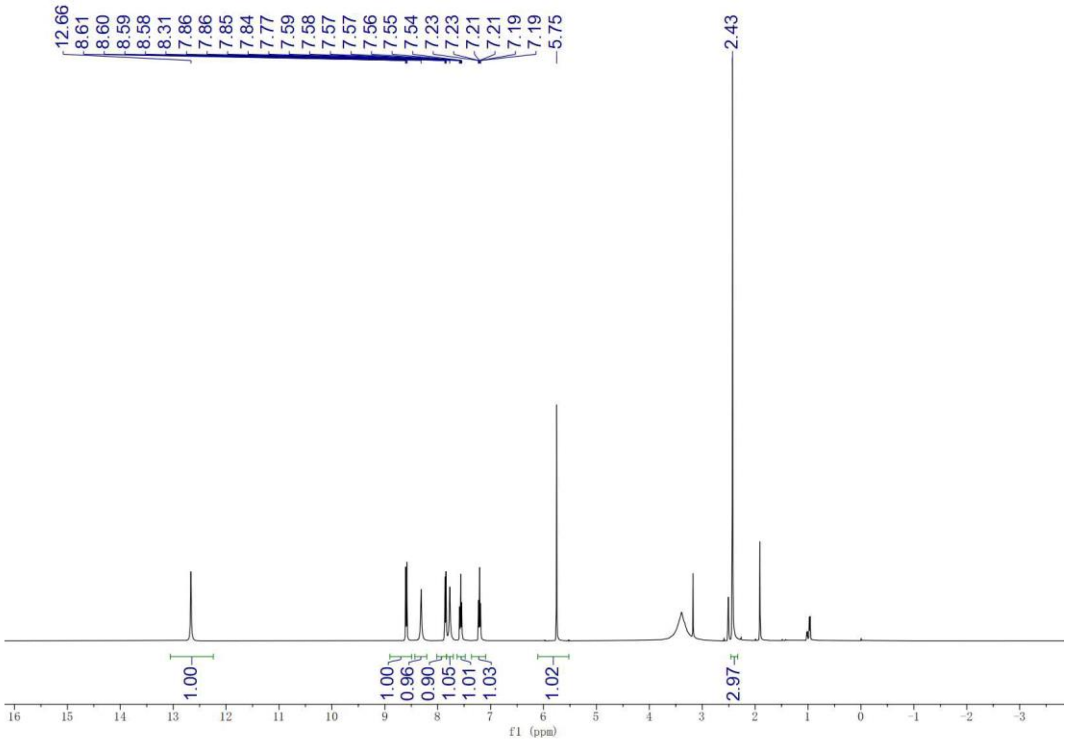

Figure S4 The ^{13}C NMR spectrum of 2-pyruvoylaminobenzamide (**2**) (100 MHz, DMSO-d_6)

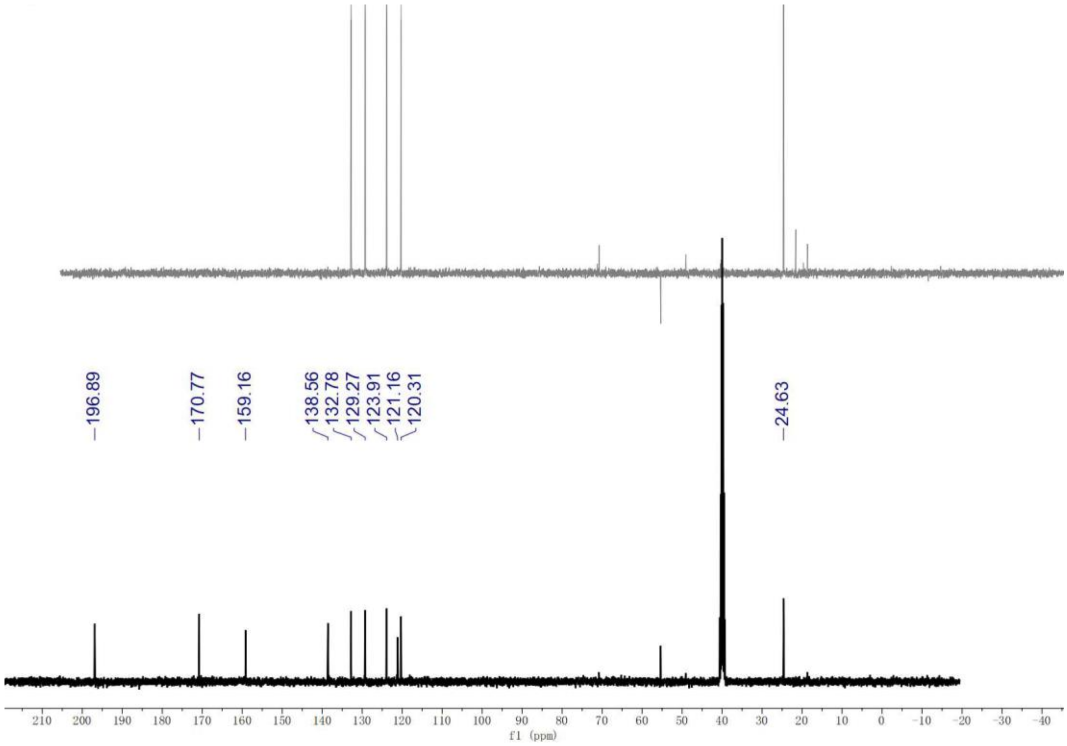

Figure S5 The ^{1}H NMR spectrum of 4-hydroxybenzaldehyde (**3**) (400 MHz, MeOD)

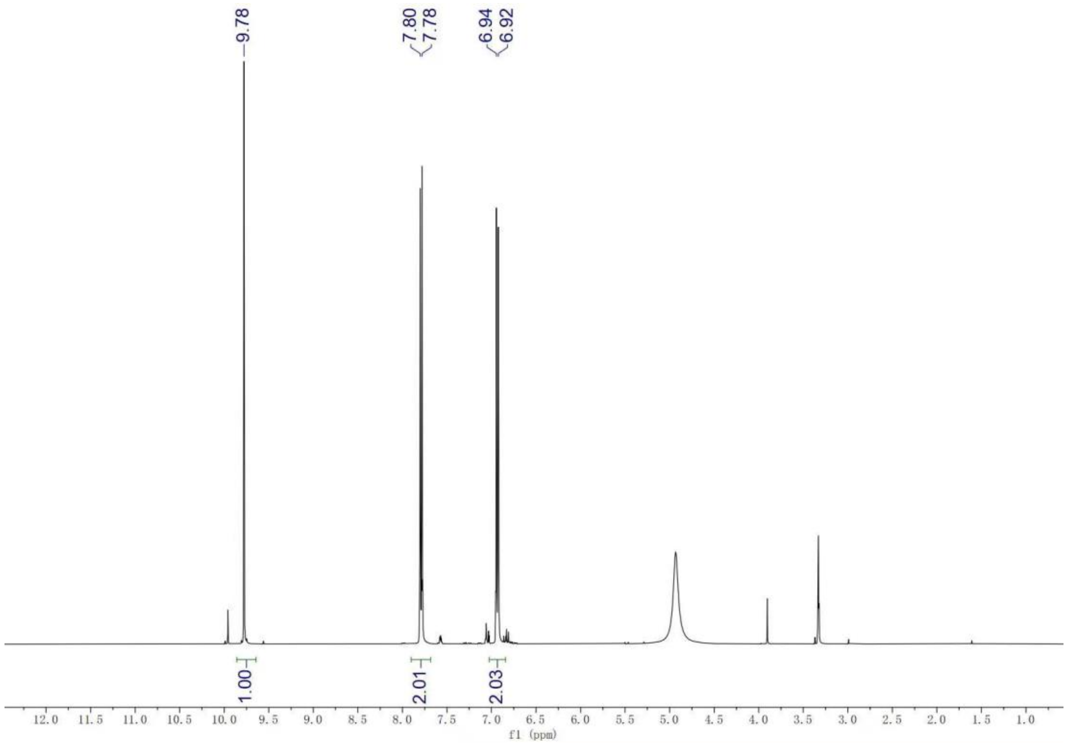

Figure S6 The ^{13}C NMR spectrum of 4-hydroxybenzaldehyde (**3**) (100 MHz, MeOD)

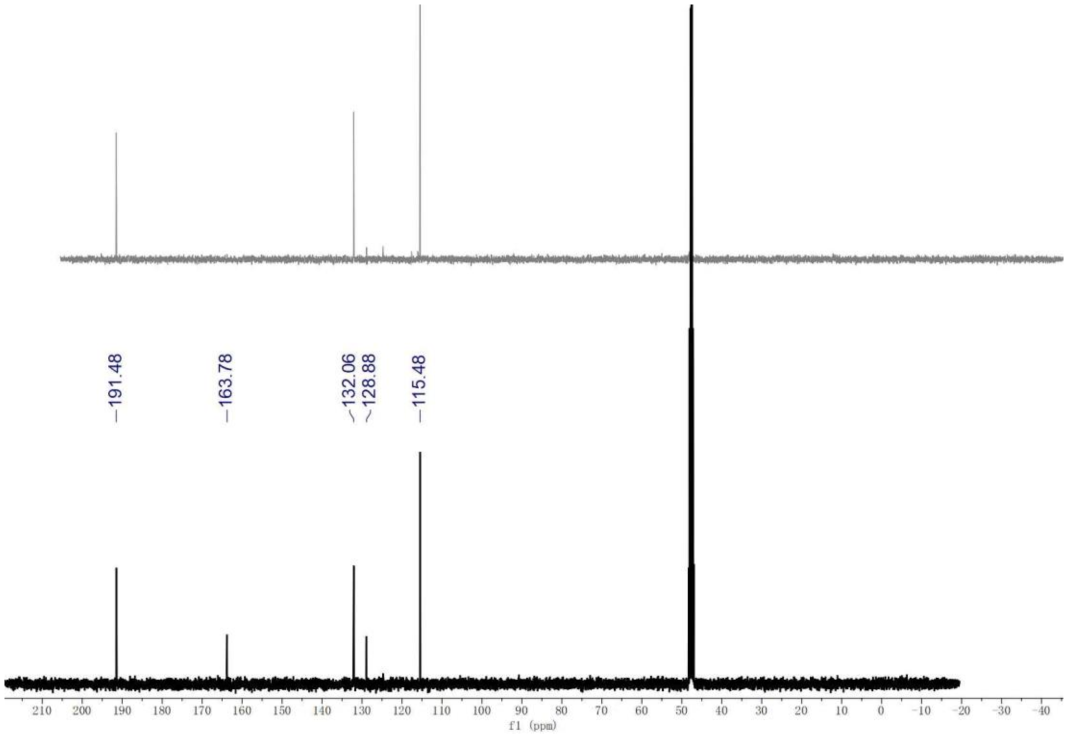

Figure S7 The ^{1}H NMR spectrum of stigmasta-7,22-diene-3β,5α,6α-triol (**4**) (400 MHz, DMSO-d_6)

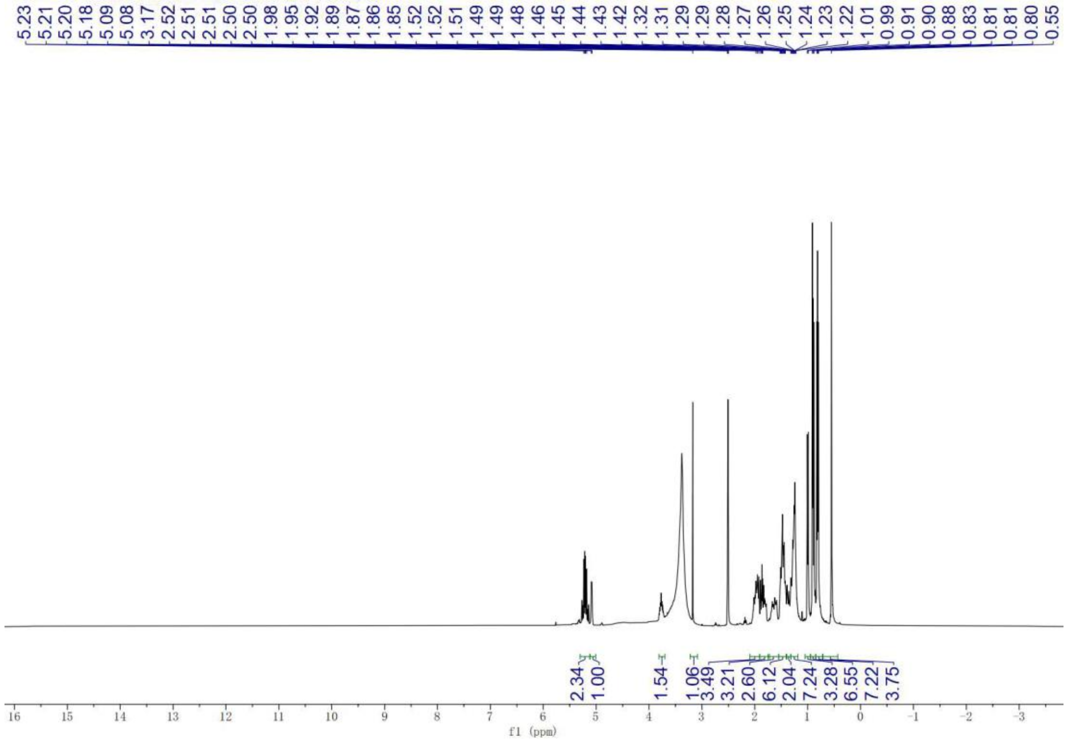

Figure S8 The ^{13}C NMR spectrum of stigmasta-7,22-diene-3β,5α,6α-triol (**4**) (100 MHz, DMSO-d_6)

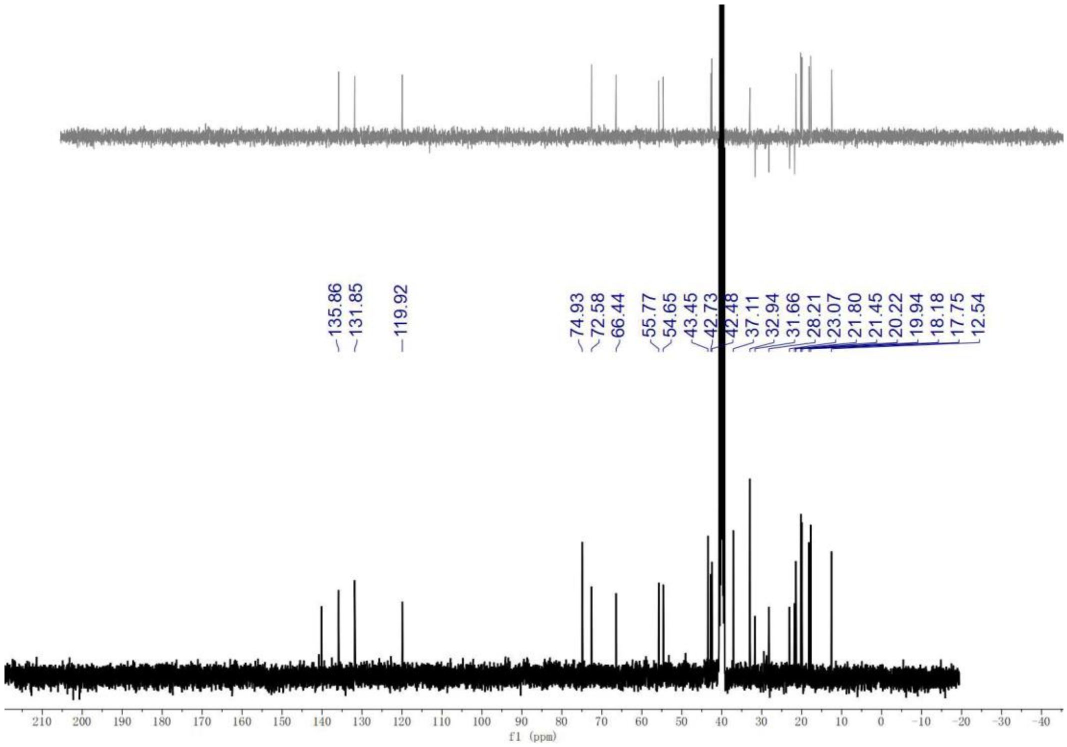

Figure S9 The ^{1}H NMR spectrum of (2*S*,5*R*)-2-ethyl-5-methylhexanedioic acid (**5**) (600 MHz, MeOD)

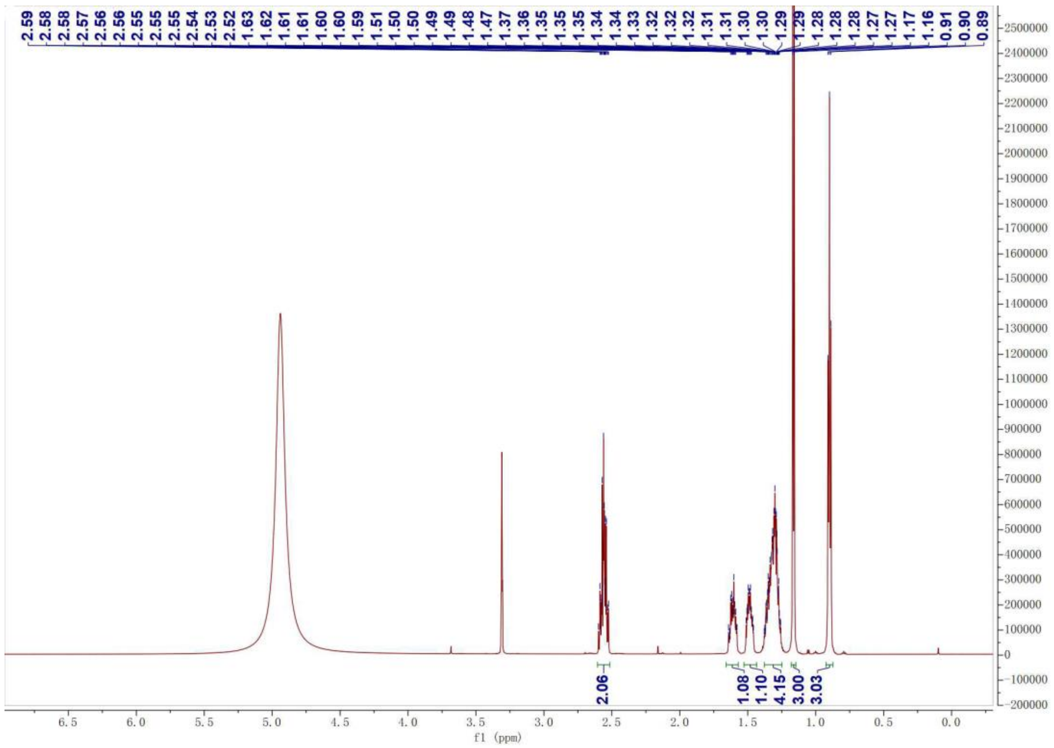

Figure S10 The ^{13}C NMR spectrum of (2*S*,5*R*)-2-ethyl-5-methylhexanedioic acid (**5**) (150 MHz, MeOD)

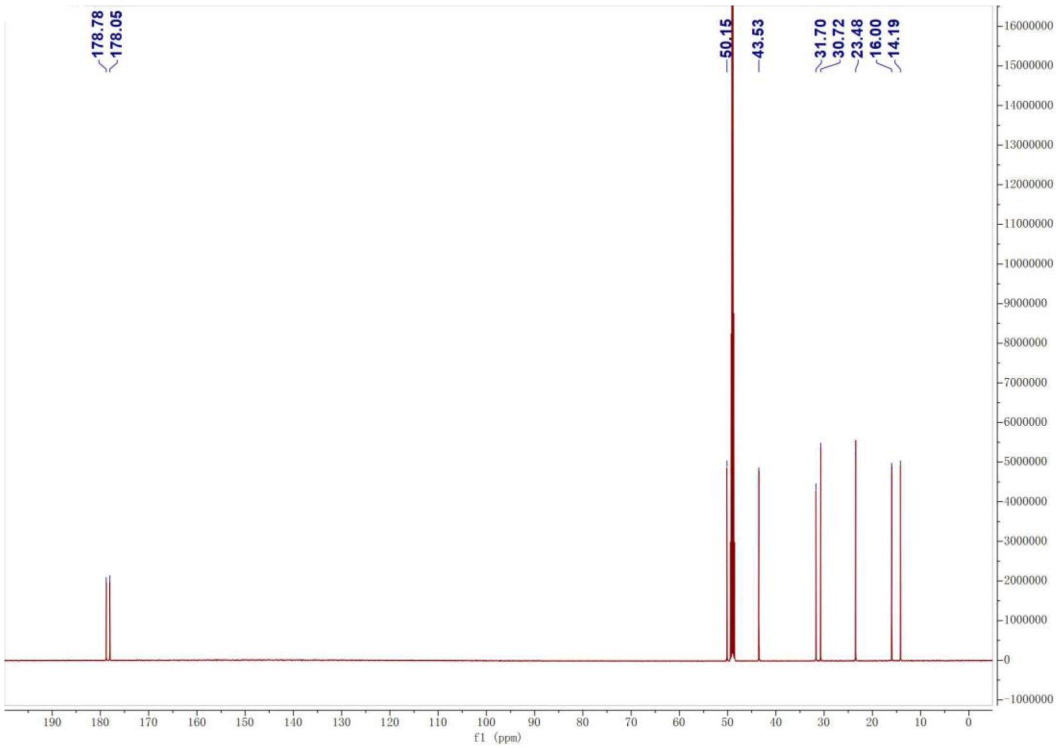

Figure S11 The ESIMS/MS of (2*S*,5*R*)-2-ethyl-5-methylhexanedioic acid (**5**)

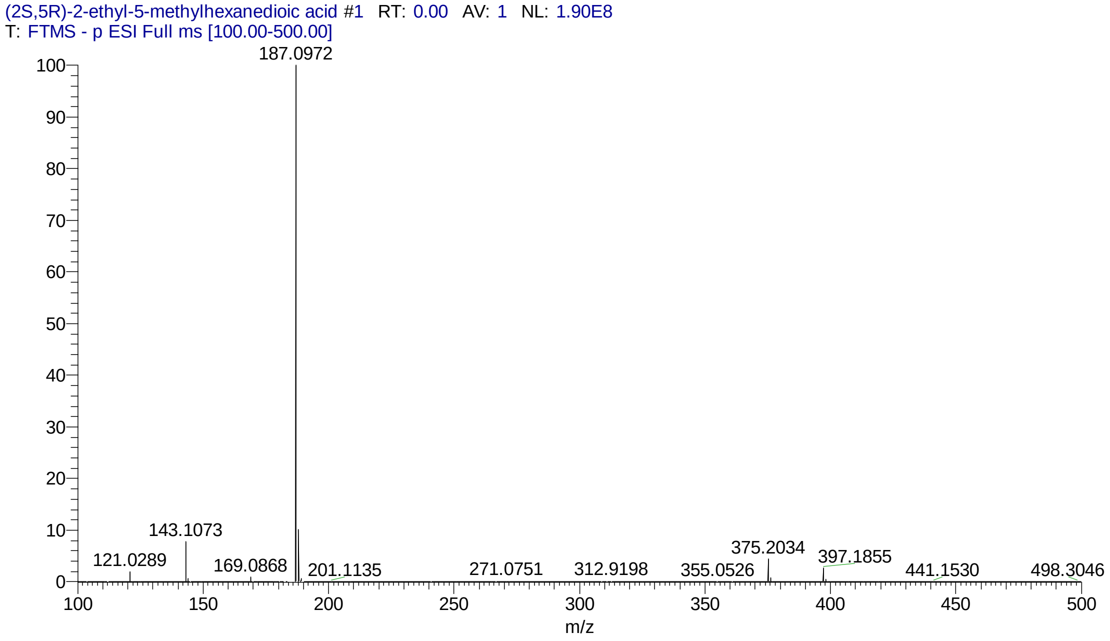

Figure S12 The HPLC chromatogram of (2*S*,5*R*)-2-ethyl-5-methylhexanedioic acid (**5**)

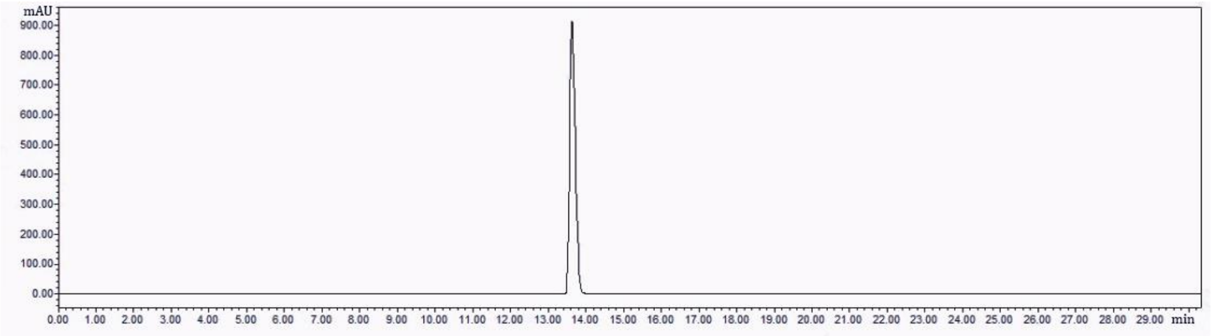

Note: High-performance liquid chromatography (HPLC) analysis for compound **5** was conducted using a Waters e2695-ELS detector (Waters Corporation, Massachusett, USA) with an Agilent Zorbax SB-C_{18} column (4.6 mm × 250 mm,5 μm, Agilent Technologies Inc., California, USA). A mobile phase of acetonitrile-water (gradient elution, 80%→5%, 0–40 min) at a flow rate of 1 mL/min was adopted. The sample injection volume was 10 μL. The air was used as the atomizing gas with the gas pressure set to 30 psi. The nebulizer was set in Cooling mode, and the drift tube temperature was 60℃, with a carrier gas flow rate of 2.38 SIPM/min.
